# Deciphering tumor microenvironment: CXCL9 and SPP1 as crucial determinants of tumor-associated macrophage polarity and prognostic indicators

**DOI:** 10.1186/s12943-023-01931-7

**Published:** 2024-01-13

**Authors:** Xinming Su, Chenhao Liang, Ruixiu Chen, Shiwei Duan

**Affiliations:** 1 Key Laboratory of Novel Targets and Drug Study for Neural Repair of Zhejiang Province, School of Medicine, Hangzhou City University, Hangzhou, Zhejiang China; 2 Department of Clinical Medicine, Hangzhou City University, Hangzhou, Zhejiang China

**Keywords:** Tumor microenvironment, Tumor-associated macrophages, Biomarkers, CXCL9, SPP1, Prognosis

## Abstract

The tumor microenvironment (TME) is an intricate system comprised of tumor cells and the surrounding cellular and non-cellular components, exerting a pivotal influence on the initiation and progression of tumors. Exhibiting dynamic and diverse compositions as well as functional states across various tumors and patients, a profound comprehension of its specific internal interactions is indispensable for formulating efficacious anti-cancer treatment strategies. Extensive interactions among various immune cell types within the TME are well-documented, with their phenotypes and abundances closely linked to clinical prognoses. TME research is progressing towards greater complexity and precision, yet, to date, no representative TME biomarkers suitable for clinical applications have been definitively identified and validated. In a recent study, the collaborative actions of CXCL9 and SPP1 (CXCL9:SPP1) were found to collectively dictate the polarity of tumor-associated macrophages (TAMs) within the TME, exerting profound effects on tumor progression and treatment responses. The mutually exclusive expression of CXCL9:SPP1 in the TME not only governs TAM polarity but also exhibits strong correlations with immune cell profiles, antitumor factors, and patient outcomes, significantly influencing prognosis. This article consolidates the significance and prospects of CXCL9:SPP1 as a novel indicator for tumor development and prognosis, while also proposing future research directions and addressing potential challenges in this promising field.

**Supplementary Information:**

The online version contains supplementary material available at 10.1186/s12943-023-01931-7.

## To the editor

The tumor microenvironment (TME) constitutes a distinctive milieu encompassing a multitude of cell types and secreted factors enveloping tumor cells [1]. Genome instability and mutation represent crucial attributes within the TME [2]. Given the multifaceted heterogeneity and diversity observed across various tumor types and among individual patients [2], this particular characteristic exhibits notable variability. Nevertheless, in a broad context, components within the TME exhibit greater genetic stability compared to tumor cells, which frequently undergo extensive mutations, rendering them more amenable targets for therapeutic intervention [1]. Deciphering the intricate components of the TME ecosystem and comprehending the nuanced interactions among them yields invaluable insights into the regulatory mechanisms governing tumor progression, drug resistance, and treatment responses.

As pivotal players in the immune system, macrophages are widely distributed across various tissues. Their functional roles within the TME guide the coevolution of the cancer ecosystem throughout tumor development, metastasis, and therapeutic responses [3]. Within the TME, tumor-associated macrophages (TAMs) emerge as the predominant immune cell subpopulation [3]. TAMs, based on their phenotype and function, are subdivided into two major subtypes: M1 and M2 in long stretches [4]. M1 macrophages excel in tumor cell killing and resistance against pathogen invasions, whereas M2 macrophages predominantly promote tumor progression and immune suppression, epitomizing a narrower interpretation of TAMs [4]. Recognizing the distinctive role of TAMs in the TME, macrophage-directed tumor therapeutics have gained momentum, encompassing strategies like targeted polarization and TAM clearance [4]. However, in light of the swift progress in single-cell sequencing technology throughout the last decade, there has been a noteworthy revelation regarding the extensive heterogeneity of TAMs in terms of morphology, function, and cell surface marker expression. This heterogeneity extends to diverse aspects such as transcriptome composition, epigenetic profiles, metabolic characteristics, multi-omics traits, and more, as highlighted in reference [5]. Consequently, the oversimplified M1/M2 model, ill-suited for the nuanced reality, stands as an obsolete framework. Instead, embracing more sophisticated models, such as functional spectrum models, becomes imperative for accurately delineating macrophage subpopulations and establishing a more precise correlation between the phenotype and function of TAMs, as discussed in reference [5]. In conclusion, the complexity of the TME and its underlying regulatory mechanisms warrant continued investigation.

Substantial research has illuminated the regulatory roles of CXCL9 and SPP1 (CXCL9:SPP1) individually in cancer. C-X-C motif chemokine ligand 9 (CXCL9), also known as monokine induced by gamma interferon (MIG), belongs to the ELR-negative CXC chemokine subfamily [6]. Mainly synthesized by macrophages, CXCL9 co-localizes with CCL4 or CXCL13 in LAG3^+^ T cells. It plays a pivotal role in immune cell activation and signaling associated with inflammatory responses, fostering a “hot” TME conducive to immune responses and bolstering the body’s anti-tumor capabilities [7]. Secreted phosphoprotein-1 (SPP1), also known as osteopontin (OPN), functions as a crucial adhesion protein and cytokine capable of upregulating interferon-γ and interleukin-12 expression [8]. Its distinctive structural properties and integrin-binding prowess render SPP1 an effective signaling molecule [8]. Notably, SPP1 is specifically expressed in macrophages and fosters macrophage polarization, migration, sustained activation, and impacts the cytokine profile of macrophages [8]. Furthermore, some recent studies have increasingly highlighted the potential synergistic interactions between CXCL9, SPP1, and TAMs [9, 10].

In a groundbreaking study recently published in *Science*, Pittet et al. unveiled a pioneering population-centric single-cell RNA sequencing (scRNA-seq) approach [11]. This transformative shift redirects the focus from individual cells to the entire tumor as the statistical unit of analysis. This innovative methodology aims to unravel the intricate complexities of the TME and discern the underlying principles governing its composition and its profound implications on disease outcomes [11]. Their research highlights the mutually exclusive expression of CXCL9:SPP1 in TAMs, a crucial determinant of whether these TAMs adopt an anti-tumor or pro-tumor phenotype. Significantly, CXCL9:SPP1 expression exhibits close associations with the expression patterns and abundance of other cell types, establishing it as a novel indicator for assessing tumor progression and therapeutic efficacy.

Firstly, Mikael J. Pittet et al. meticulously assembled a cohort comprising 51 patients with head and neck squamous cell carcinoma (HNSCC), encompassing diverse tumor characteristics such as primary tumors, locally recurrent tumors, and distant metastases, thus enhancing the reliability and generalizability of the study outcomes. Notably, the scRNA-seq technology employed eschewed pre-screening or cell enrichment procedures in the obtained tissues, ensuring the acquisition of unaltered and comprehensive data. In their analysis, the authors leveraged Seurat’s FindCluster method to scrutinize a substantial pool over 180,000 cells. Impressively, the manual annotation process undertaken by the authors spanned various resolutions, encompassing main compartments, major cell types, major immune cell types, and minor cell states, all achieved without reliance on predefined cell markers. Simultaneously, the authors innovatively enhanced the scRNA-seq data analysis by introducing individual-based modes, treating each patient’s tumor as an independent statistical unit rather than concentrating solely on individual cells. This holistic approach facilitated a thorough examination of diverse cell types and their gene expressions within the TME, coupled with histological analysis to yield crucial spatial information. Consequently, the study yielded valuable insights into the cellular heterogeneity of the TME across different patients and illuminated its impact on disease progression. Their comprehensive analysis identified 1,189 genes predicated on the expression profiles within tumor tissues and validated the consistency of gene expression across distinct groups by integrating additional independent datasets. Intriguingly, non-tumor constituents, notably mast cells and TAMs, retained autonomous prognostic significance. Of utmost significance, the CXCL9:SPP1 ratio within TAMs exhibited significant associations with multiple prognostic parameters across all patient cohorts. Consequently, the authors embarked on an exhaustive exploration of CXCL9:SPP1 in TAMs. Through scRNA-seq analysis and comprehensive histological assessments of whole tumors, the authors discovered that TAM abundance alone failed to serve as an independent prognostic indicator. Nevertheless, within the spectrum of TAM states, the reciprocal expression of CXCL9:SPP1 demonstrated an antagonistic relationship capable of predicting diverse patient prognoses. This CXCL9:SPP1 expression ratio was termed the “polarity” of TAMs, underscoring its superior utility compared to the commonly used M1 and M2 markers, as it provides precise and clinically relevant information. Importantly, this groundbreaking discovery transcends the confines of HNSCC and holds true in other solid malignancies.

Furthermore, Pittet et al. conducted a comprehensive classification of 52 HNSCC samples based on the polarity of CXCL9:SPP1 in TAMs, correlating it with TME variables like cell type abundance and gene expression. The results unveiled a striking association between TAM polarity defined by CXCL9:SPP1 and anti-tumor immune responses within the TME. Samples with low CXCL9:SPP1 ratios exhibited gene expression signatures conducive to tumor promotion within the TME, whereas those with high CXCL9:SPP1 ratios displayed the opposite trend. This observation underscores the existence of a finely orchestrated communication network operating within the complex TME milieu, with CXCL9:SPP1 potentially serving as a pivotal regulatory axis influencing tumor development. The opposing expression patterns of CXCL9:SPP1 originate from distinct microenvironments that house CXCL9^+^ and SPP1^+^ TAMs: an environment rich in IFN-γ fosters CXCL9 expression, while hypoxic conditions promote SPP1 expression. Further insights were gleaned from RNA in situ hybridization and immunofluorescence analyses of tumor tissues, which illustrated the spatial clustering of CXCL9^+^ or SPP1^+^ tumor cells and TAMs, particularly at the interface of CXCL9^+^ and SPP1^+^ TAM distributions. Furthermore, univariate and bivariate analyses preliminarily confirmed distinct spatial distribution patterns for CXCL9^+^ and SPP1^+^ TAMs across various cancer types.

Accurately characterizing the TME is crucial in cancer treatment [1]. However, owing to the TME’s intricate specificity and heterogeneity, many aspects of its behavior remain the subject of ongoing debate [2]. Thus far, a slew of high-quality studies has progressively unveiled the intricate interactions between cancer cells, non-cancerous cells, and the cellular matrix constituting the TME. Consequently, more nuanced concepts like subTME have emerged to elucidate the non-random heterogeneity observed across different temporal and spatial dimensions [12]. Looking ahead, research on the TME necessitates greater resolution and extended temporal scopes. The widespread adoption of single-cell multi-omics sequencing technology stands as a vital tool to refine our understanding of the communication dynamics among cancer cells, other cell types, and the extracellular matrix within the TME. This approach also holds promise in elucidating the evolution of the TME during cancer progression and the evolving biological characteristics that emerge under specific treatment conditions.

Translating the TME’s complex characteristics into clinical applications requires simplifying these complexities. This transformation necessitates the identification of dependable markers that encapsulate the overall biological behavior of the TME. These markers should ideally possess characteristics such as easy accessibility, stability, high specificity, and a close association with both physiological conditions in the body and the onset and progression of cancer. Within the TME, TAMs engage in intricate interactions with cancer and stromal cells, playing a pivotal role in shaping the attributes of various cancers [3]. The study conducted by Pittet et al. primarily focused on HNSCC, delving deep into the relationship between CXCL9:SPP1 expression within the TME and clinical prognosis. It underscored the potential of the CXCL9:SPP1 gene ratio as a clinical and prognostic marker for cancer treatment, effectively representing TAM polarity. Nevertheless, several crucial and thought-provoking queries persist regarding the conclusions drawn from these studies. Primarily, the authors narrowed their focus to CXCL9:SPP1 based on gene expression within a limited number of cells, a factor that may introduce certain limitations. Is there a simpler intermediary between CXCL9:SPP1 capable of directly substituting the ratio highlighted in the article? Furthermore, the primary investigation concentrated on HNSCC, prompting the need for additional validation of its applicability in other cancer types. It is noteworthy that preceding research in cancers such as ovarian cancer (OC) [13] and infiltrative basal cell carcinoma (iBCC) [10] failed to identify mutually exclusive expression patterns of CXCL9:SPP1, even instead revealed a codirectional relationship. This implies a likelihood of distinct biomarkers for various cancers and subtypes, underscoring the necessity for further exploration and validation in subsequent studies. This could be just the initial step in unraveling the foundational regulatory principles underpinning the complexity of the TME. Future research avenues can broaden the scope to encompass diverse cancer types, delve into distinct cell subpopulations, and pinpoint core regulatory genes to identify more dependable biomarkers and prognostic models. Furthermore, efforts to validate their presence in easily accessible samples such as blood, cerebrospinal fluid, and tissue fluid should be intensified, facilitating the clinical translation of such biomarkers.

**Fig. 1:**
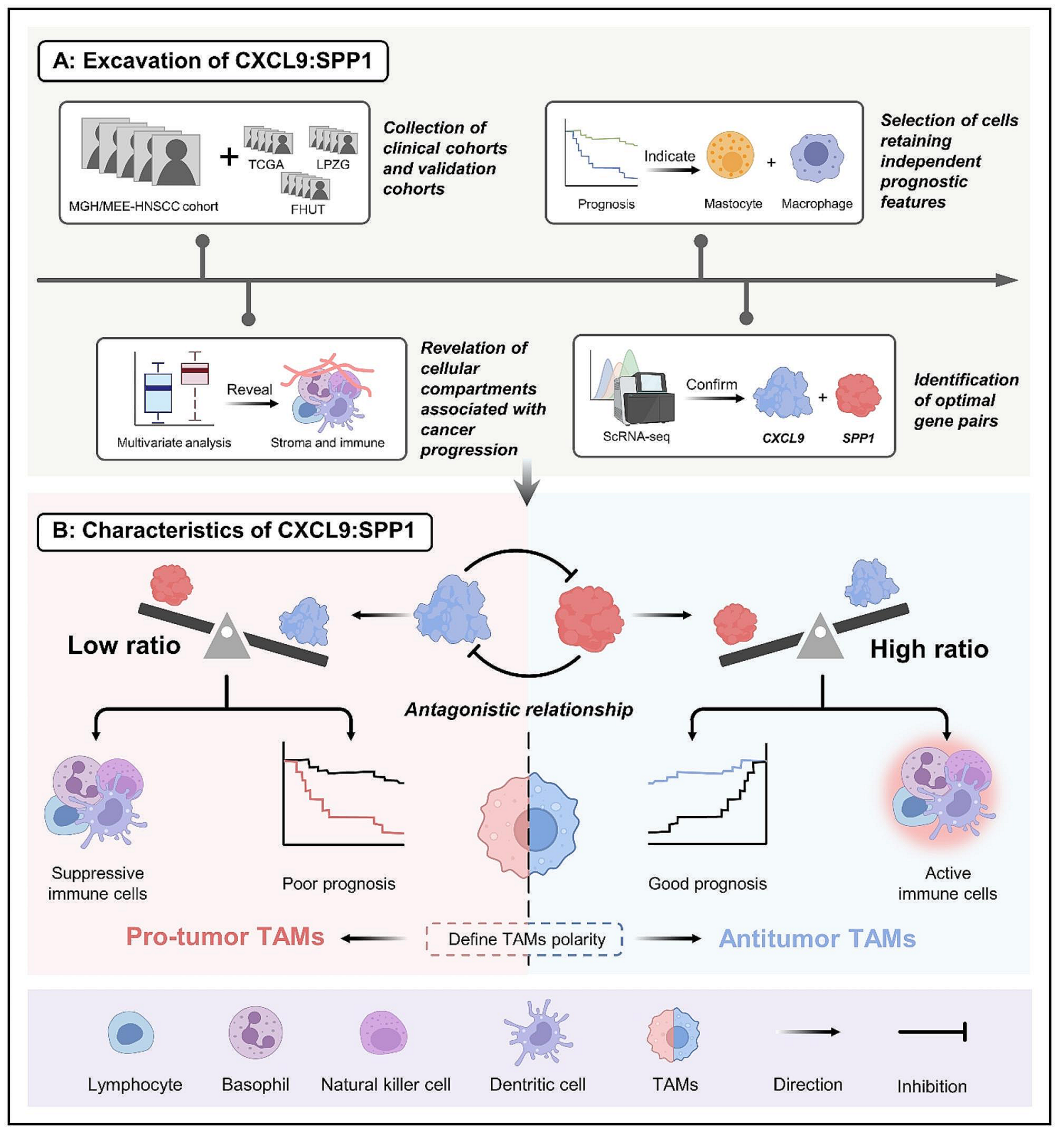
CXCL9:SPP1 polarity as an indicator of TME immune activity and prognosis in cancer patients. The CXCL9:SPP1 polarity could serve as an indicator of immune activity within the TME and may hold significance for patient prognosis. A low CXCL9:SPP1 ratio defining TAM polarity suggests a TME enriched with immune activation and anti-tumor factors, pointing towards a promising prognosis. Conversely, a low CXCL9:SPP1 ratio characterizing TAM polarity indicates the prevalence of immunosuppressive and tumor-promoting factors within the TME, correlating with a poorer prognosis. CXCL9:SPP1, CXCL9 and SPP1; TAMs, tumor-associated macrophages; TME, tumor microenvironment

In summary, Pittet et al.‘s innovative study hints at the existence of a fundamental, underlying logic amidst the complexity of the TME (Fig. 1). As research advances, this effective assessment methodology is expected to demonstrate its unique advantages, including specificity and feasibility, in clinical translation. Clinicians stand to gain from employing this approach to predict a patient’s probable response to different therapies and formulate personalized treatment strategies tailored to each patient’s distinct tumor characteristics.

## Electronic supplementary material

Below is the link to the electronic supplementary material.

Supplementary Material 1

## Data Availability

Not applicable.

## Abbreviations

CXCL9: C-X-C motif chemokine ligand 9
CXCL9:SPP1: CXCL9 and SPP1
HNSCC: Head and neck squamous cell carcinoma
iBCC: Infiltrative basal cell carcinoma
MIG: Monokine induced by gamma interferon
OC: Ovarian cancer
OPN: Osteopontin
scRNA-seq: Single-cell RNA sequencing
SPP1: Secreted phosphoprotein-1
TAMs: Tumor-associated macrophages
TME: Tumor microenvironment
