# Supplementary material for: Deciphering tumor microenvironment: CXCL9 and SPP1 as crucial determinants of tumor-associated macrophage polarity and prognostic indicators
Source: Mol Cancer. 2024 Jan 13;23:13. doi: 10.1186/s12943-023-01931-7 (PMC10790255; doi:10.1186/s12943-023-01931-7)
Supplement: Supplementary file 1 — Supplementary Material 1 [file 12943_2023_1931_MOESM1_ESM.docx]

**Full uncropped Gels and Blots image(s)**

Not applicable.
